# Starvation from Enlargement of the Glands at the Cardiac Orifice of the Stomach

**Published:** 1844-03-23

**Authors:** 


### STARVATION FROM ENLARGEMENT OF THE GLANDS AT THE CARDIAC ORIFICE OF THE STOMACH.

In this singular case the enlargement of the glands in question prevented food from passing into the stomach, thereby inducing extreme marasmus and death.—Mrs. Kerrit, aged fifty-three, for twelve months, or longer, had felt a difficulty in deglutition, the sensation being as though the food were stopped in its passage to the stomach; when it returned it was streaked with blood. Under these circumstances she came into the Colchester hospital, where she remained two or three weeks, and although, by taking fluid aliment frequently and in small quantities, she occasionally rallied, yet, it appearing evident that no permanent benefit could be received, she was removed home, and died in about a week or ten days.

The liver was enlarged, and a portion of the edge of the left lobe adhered to the cardiac orifice of the stomach. There were two or three tubercles in the liver, each about an inch in diameter, and of the consistence of cartilage. The mucous membrane passing from the œsophagus through the cardiac orifice of the stomach was thickened, and for two or three inches into the stomach it appeared to be raised from the level of the healthy membrane, so as to define the extent of the altered tissue. The glands at the cardiac orifice were enlarged.

It seems that the solid articles of diet accumulated at the seat of the stricture caused by the thickened membrane and the enlarged glands, the œsophagæal muscular fibres being thereby neutralized in their actions. When the food arrived at the constriction it would not pass beyond, and as hypertrophied parts are increased in sensibility, the irritation would, I apprehend, produce a contraction of the œsophagæal muscular fibres, beginning at the stricture and proceeding upwards; hence the food was regurgitated by mouthfuls, as it was swallowed!—*London Lancet.*

